# Comparison of Biological Properties and Clinical Application of Mesenchymal Stem Cells from the Mesoderm and Ectoderm

**DOI:** 10.1155/2023/4547875

**Published:** 2023-06-10

**Authors:** Zhenning Wang, Meng Huang, Yu Zhang, Xiaoxia Jiang, Lulu Xu

**Affiliations:** ^1^Medical School of Chinese PLA, Beijing 100853, China; ^2^Department of Orthodontics, The First Medical Center, Chinese PLA General Hospital, Beijing 100853, China; ^3^Beijing Institute of Basic Medical Sciences, Beijing 100850, China

## Abstract

Since the discovery of mesenchymal stem cells (MSCs) in the 1970s, they have been widely used in the treatment of a variety of diseases because of their wide sources, strong differentiation potential, rapid expansion in vitro, low immunogenicity, and so on. At present, most of the related research is on mesoderm-derived MSCs (M-MSCs) such as bone marrow MSCs and adipose-derived MSCs. As a type of MSC, ectoderm-derived MSCs (E-MSCs) have a stronger potential for self-renewal, multidirectional differentiation, and immunomodulation and have more advantages than M-MSCs in some specific conditions. This paper analyzes the relevant research development of E-MSCs compared with that of M-MSCs; summarizes the extraction, discrimination and culture, biological characteristics, and clinical application of E-MSCs; and discusses the application prospects of E-MSCs. This summary provides a theoretical basis for the better application of MSCs from both ectoderm and mesoderm in the future.

## 1. Introduction

As an important member of the stem cell family, mesenchymal stem cells (MSCs) are widely distributed and easy to extract and culture and have self-replication ability and strong differentiation potential. MSCs also have the ability to migrate to damaged tissues and regulate the immune response according to the microenvironment, which is why they are being increasingly applied in tissue engineering and clinical research.

MSCs are derived from the mesoderm and ectoderm in early development. Leucht et al. [1] proposed that damaged tissue from different germ layers will recruit MSCs from the corresponding germ layers for repair. Moreover, the proliferation and differentiation abilities of MSCs from different tissues are also different [2]. This paper comprehensively compares the biological characteristics and clinical application of mesenchymal stem cells from the mesoderm and ectoderm, as well as the possible development direction in the future.

## 2. Basic Introduction

### 2.1. Mesoderm-Derived MSCs (M-MSCs)

A rich source of M-MSCs is an important basis for their extensive research and application. Bone marrow is an important source of MSCs. In addition to bone marrow, M-MSCs also exist in various tissues and organs. In 2000, human umbilical cord blood stem cells were first reported [3]. Subsequently, in 2001, adipose tissue and synovium were also proven to be rich sources of M-MSCs [4, 5]. The extraction method of M-MSCs, in short, includes separating various tissues, digesting the tissues to obtain cells, culturing the cells for 3 to 5 days, discarding nonadherent cells, and continuously culturing adherent cells to the desired passage [6].

### 2.2. Ectoderm-Derived MSCs (E-MSCs)

**Figure 1:**
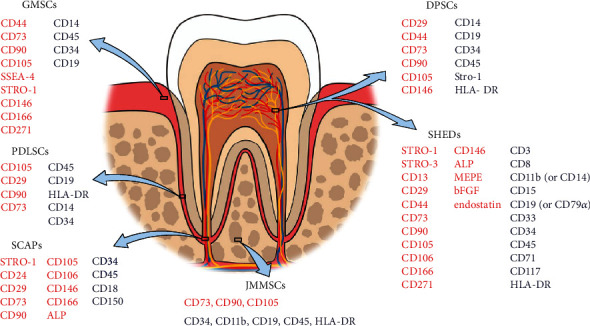
Sources and markers of some representative E-MSCs (DPSC-dental pulp stem cells, SCAP-apical dental papilla stem cells, SHED-deciduous dental pulp stem cells, PDLSC-periodontal ligament stem cells, GMSC-gingival mesenchymal stem cells, and JMMSC-jaw marrow-derived mesenchymal stem cells. Red indicates positively expressed while blue indicates negatively expressed surface markers).

E-MSCs are mainly divided into three types: osteogenic (T.Q. [7]), odontogenic, and olfactory mucosal. Odontogenic stem cells include dental pulp stem cells (DPSCs), dental follicle stem cells (DFSCs), apical dental papilla stem cells (SCAPs), deciduous dental pulp stem cells (SHEDs), and periodontal ligament stem cells (PDLSCs). Sources and markers of odontogenic stem cells are listed in Figure 1. DPSCs were first isolated from adult dental pulp in 2001 [8]. DFSCs were isolated and identified from the dental sac of human third molars by Morsczeck et al. [9] in 2005. Sonoyama et al. [10] first found and identified SCAPs from the apical papilla of extracted third molars in 2006. SCAPs come from developing tissues. Therefore, SCAPs may have better regeneration potential than other mature tissues. Miura et al. (S. Shi) first discovered SHEDs in 2003. SHEDs can differentiate into a variety of cell types, including nerve cells, adipocytes, and odontoblasts. In addition, after injection into the dentate gyrus of the mouse hippocampus, SHEDs can differentiate into neural tissue and express neuronal and glial markers, indicating that SHEDs can be used for dental pulp and nerve regeneration. PDLSCs were isolated and identified from the surface of tooth roots by Seo et al. [11] in 2004. In 2009, Zhang et al. [12] first reported the isolation, characterization, and immunomodulatory properties of gingival mesenchymal stem cells (GMSCs) and found that GMSCs can inhibit the proliferation of peripheral blood monocytes induced by phytohemagglutinin. Olfactory mucosal stem cells (OMSCs) are MSCs isolated from the olfactory mucosal epithelium. Studies have shown that MSCs may play a better role in promoting the formation of the myelin sheath in the central nervous system and in repairing nerve injury than MSCs from other tissues [13].

## 3. Extraction, Discrimination, and Culture

**Figure 2:**
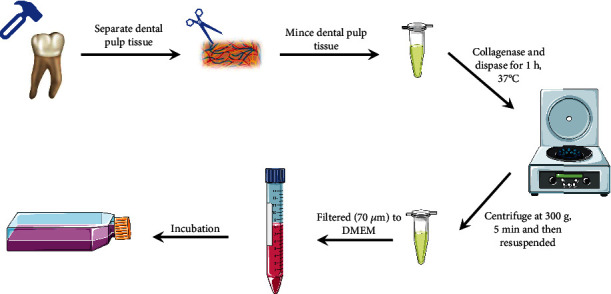
Typical extraction process of DPSCs of human.

Theoretically, MSCs can be isolated from all tissues. For example, M-MSCs mainly come from the bone marrow, adipose tissue, placenta and human umbilical cord blood, while E-MSCs mainly come from the dental pulp, the jaw, the frontal bone, the periodontal ligament, the gingiva, and the dental papilla. Han et al. [6] summarized the extraction, identification, and culture methods of several common M-MSCs. For comparison, in Table 1, the authors summarized the extraction, identification, and culture of several common E-MSCs. In Figure 2, the extraction process of DPSCs is described in the form of a schematic.

## 4. Biological Properties

Regardless of the source of MSCs, they all show some common characteristics, such as fibroblast-like morphology, cell surface markers, cell proliferation ability, and multidirectional differentiation potential. However, the biological properties of MSCs from different tissues or the same kind of MSCs under different conditions are slightly different.

### 4.1. Cell Proliferation Ability

According to Miura et al. (S. Shi), SHEDs have a higher proliferation rate and population doubling efficiency than DPSCs and bone marrow mesenchymal stem cells (BMMSCs). In addition, SHEDs are separated from deciduous teeth, so they can be easily obtained without ethical considerations. The transition from deciduous teeth to permanent teeth is a unique dynamic process. The dental pulp of deciduous teeth already exists before birth, indicating that these stem cells are not affected or are less affected by environmental factors than other stem cells [14]. Akintoye et al. [35] compared MSCs from maxillofacial and lower limb bone marrow, and the results showed that the proliferation rate of jaw bone marrow MSCs from maxillofacial-bone marrow was faster than that of iliac bone marrow MSCs from lower limb bone marrow. Moreover, compared with long bone marrow MSCs, jaw bone marrow MSCs showed stronger proliferation and antiapoptotic potential [7, 20]. In embryology, the iliac bone and long bone are derived from the mesoderm, while the jaw is derived from the ectodermal neural crest. Interestingly, studies have shown that cells expressing neural crest markers have stronger stem cell properties and stronger proliferation potential than M-MSCs [2]; thus, they contribute to the survival of MSCs under hypoxia after transplantation [21, 36].

### 4.2. Multidirectional Differentiation

Regardless of the kind of tissue source, MSCs have multidirectional differentiation potential. Multidirectional differentiation potential is one of the important characteristics of MSCs. The differentiation trend of different tissue sources is also different. The jaw develops from the neural crest cells of ectoderm [37], while the mesenchymal cells of mesoderm develop into the long bones of the limbs [38], and the osteogenic processes experienced by the two bone tissues are also different in the development process [37]. In some diseases, such as osteoporosis and hyperthyroidism, the involvement of the long bone is significantly higher than that of the jaw [39]. Some studies have shown that compared with bone marrow MSCs of long bones of limbs, jawbone marrow MSCs have stronger osteogenic activity. This may be due to the high expression of BMP-4, nestin, and other neural crest-related genes in jawbone marrow MSCs at the transcriptional level [2], which is consistent with the research results of Aghaloo et al. [40].

Central and peripheral nerve injuries are difficult to treat, because the ability of the nervous system to repair damaged cells and tissues is limited. In this regard, E-MSCs have obvious advantages because they can differentiate into neuron-like cells and express neuronal markers, such as STRO-1, nestin, c-FOS, GFAP, and *β* III-tubulin [41–45]. Although these cells can differentiate into neuron-like cells, they do not further differentiate into functional neurons [41]. On this basis, Kiraly et al. found that simultaneous activation of PKC and cAMP can induce hDPSCs to differentiate into functional neurons [43].

At present, research in the field of nerve regeneration mainly focuses on DPSCs and SHEDs [19, 46, 47]. This may be due to the relationship between their tissue origins. In the process of tooth development, the tooth germ is composed of an enamel organ, dental papilla, and dental sac. DPSCs, SHEDs, and SCAPs are derived from dental papilla; DFSCs and PDLSCs are derived from dental sac; and dental pulp is derived from dental papilla. This shows that DPSCs, SHEDs, and SCAPs are highly homologous with dental pulp at the histological level. At the same time, the extraction difficulty and conditions of SCAPs are more stringent than those of DPSCs and SHEDs. Therefore, DPSCs and SHEDs may be a possible direction for pulp regeneration and even central nerve repair in the future.

Compared with bone marrow MSCs, odontogenic MSCs are more convenient to obtain and easy to expand and preserve, have high activity, and have low immunogenicity and tumorigenicity, and these characteristics make them more conducive to clinical application [7, 48]. The multiple differentiation potential and the derivation of MSCs derived from ectodermal cells are summarized in Table 2. The difference in germ layer origin makes the phenotype of odontogenic MSCs different from that of MSCs, such as BMMSCs.

### 4.3. Age-Related Changes

As the application potential of E-MSCs has been gradually explored, the preparation of sufficient E-MSCs has become a research hotspot, but it is also one of the obstacles that hinders the clinical application of E-MSCs. The age of the MSC donor has a great impact on cell proliferation activity, differentiation potential, and paracrine effect, but the specific effect is still not clear; particularly, whether elderly patients can undergo autologous stem cell therapy is currently controversial [79].

At present, research on the age-related changes in E-MSCs mainly focuses on DPSCs, and there are relatively few studies on other stem cells. In the dental pulp of aged individuals, the proportion of cells decreases, and the proportion of fiber and collagen components gradually increases with age [80]. Mitsiadis et al. [81] showed that pulp volume gradually decreases with age due to continuous production of dentin matrix by odontoblasts, which may explain, at least in part, why DPSC extraction from permanent teeth from old donors is less efficient.

Current studies on age-related changes in the biological activity of DPSCs have shown conflicting results, with some studies showing that the proliferation and differentiation potential of MSCs are independent of age [82]. However, other studies have shown that the proliferation ability, differentiation potential, and cell surface marker expression of DPSCs are affected by age [83, 84]. Therefore, young DPSCs should be collected and preserved as soon as possible, as this may be a potential treatment for elderly patients with dental diseases in the future.

### 4.4. Immunomodulation

#### 4.4.1. Immune Regulation Mechanism of MSCs

MSCs can interact with a variety of immune cells, including T cells, dendritic cells (DCs), B cells, macrophages, neutrophils, and natural killer (NK) cells [85]. Studies have shown that the immunosuppressive effect of MSCs is mainly the result of the joint action of intercellular contact and soluble immune factors [86, 87]. Soluble immune factors, including a variety of immune regulatory factors, cytokines, and growth factors, such as prostaglandin E2 (PGE-2), indoleamine 2,3-dioxygenase (IDO), and nitric oxide (NO), can respond to immune cells and activate the immune regulation of MSCs [88, 89]. In addition, indirect or direct cell contact can also cause the immunosuppressive effects of MSCs, which are mainly mediated by programmed cell death ligand 1, programmed cell death ligand 2, and membrane-bound human leukocyte antigen [90].

MSCs can also induce chemotaxis to inflammatory sites to exert immune regulation and repair damaged cells and tissues [91, 92]. Interestingly, the immunomodulatory effect of MSCs can not only inhibit the immune response but also enhance it, and which effect they have mainly depends on the function of immunosuppressants, the types of inflammatory factors, and the state of the immune system [93]. MSCs not only respond to inflammatory cytokines but also secrete immunoregulatory molecules and participate in the regulation of the inflammatory process. For example, IDO, NO, and chemokines secreted by MSCs play key roles in MSC-mediated immune regulation [94].

#### 4.4.2. Immunomodulatory Effect of E-MSCs

E-MSCs are similar to MSCs from other tissues and can regulate the activities of different immune cells [26, 95]. The immunomodulatory activity of E-MSCs is usually activated by inflammatory cytokines produced by immune cells, which indicates that there is an interaction between E-MSCs and activated immune cells. The interactions between E-MSCs and immune cells are listed in Table 3. MSCs can have a significant impact on immune cells.

*(1) Peripheral Blood Mononuclear Cells (PBMCs)*. Peripheral blood mononuclear cells (PBMCs) are mononuclear cells in peripheral blood, including lymphocytes and monocytes. E-MSCs can inhibit the proliferation of peripheral blood monocytes through paracrine signaling [12, 17, 28, 95–97], and *γ*-interferon treatment can enhance this ability [95].

*(2) Myeloid Dendritic Cells (DCs)*. Myeloid dendritic cells (DCs) maintain and regulate the immune response by accelerating the process of antigen-specific T cells and the activation of cells in the innate immune response after DC maturation [98, 99]. Studies have shown that E-MSCs have an immunosuppressive function on DCs, which can inhibit DC maturation and differentiation through a prostaglandin E2-dependent mechanism [100, 101].

*(3) Mast Cells*. Mast cells are widely distributed around microvessels under the skin and visceral mucosa and can secrete a variety of cytokines. It has been reported that E-MSCs can inhibit the release of inflammatory cytokines by mast cell 1 (HMC-1) through a prostaglandin E2-dependent mechanism but have no effect on the proliferation of HMC-1 cells [101].

*(4) Macrophages*. Macrophages are cells with significant plasticity in the immune system [102], and they can polarize into M1 or M2 macrophages [103]. Generally, M1 macrophages have significant antibacterial properties enacted by the release of a variety of chemokines and inflammatory cytokines, while M2 macrophages can reduce inflammation and accelerate tissue repair by secreting IL-10 and nutritional factors [104]. In addition, macrophages can be cocultured with MSCs to induce M2 macrophages [27, 105]. Transplantation of DPSCs into unilateral hind limb skeletal muscle can inhibit the occurrence of sciatic nerve inflammation [106]. In specific cases, for example, lipopolysaccharide-treated PDLSCs can promote the polarization of macrophages to the inflammatory M1 phenotype [107].

*(5) T Cells*. T cells are widely distributed in animal and human tissues. Once activated, they can differentiate into helper T cell (Th) 1 and the regulatory T cell (Treg) subsets Th2, Th9, and Th17 according to the stimulation intensity and microenvironment [108, 109]. It has been proven that MSCs have a close relationship with T cells [87, 110].

MSCs secrete a large number of immunosuppressive factors, chemokines, and adhesion molecules that can effectively inhibit the proliferation, apoptosis, and differentiation of T cells [92, 111]. It has been reported that E-MSCs can inhibit T cell proliferation [24, 29, 112, 113], induce T cell apoptosis, and stimulate regulatory T cell differentiation [114]. E-MSCs induce the immunomodulatory effect of T cell apoptosis, which has an anti-inflammatory effect in vivo [115]. Interestingly, although there are few relevant studies comparing the immunomodulatory ability of MSCs from different germ layers, there is evidence that human gingival MSCs have a stronger inhibitory effect on the proliferation and Th1/Th2/Th17 differentiation of mouse CD4^+^ T cells than BMSCs [26]. Comparison of the immunomodulatory ability of MSCs from different germ layers may be a future research focus of E-MSCs.

*(6) B Cells*. B cells mainly resist and hunt down foreign pathogens by producing specific antibodies [116, 117]. At present, there are relatively few studies on the effect of E-MSCs on B cells. Kwack et al. [17] found that DPSCs can inhibit the production of immunoglobulin by B cells. However, it has also been reported that MSCs inhibit the production of antibodies by B cells depending on the intensity of inflammatory stimulation and the ratio of BMSCs to B cells [118, 119].

The immunomodulatory properties of MSCs depend on the surrounding microenvironment. Activation of E-MSCs by inflammatory factors, such as *γ*-interferon, tumor necrosis factor *α*, and interleukin-1 *β*, can significantly enhance their immunomodulatory ability [120]. Activated immune cells can upregulate the expression of MSC-related proteins [121]. Activated immune cells play a key role in inducing the immunomodulatory potential of MSCs, and there is a close relationship between these cells.

## 5. Clinical Application

Studies have proven that E-MSCs are ideal seed cells for tissue engineering. DPSCs have been used to treat severe limb ischemia, tissue defects, and bone necrosis, to regenerate skin damage caused by burns, and to generate liver, nerve, skeletal muscle, blood vessels, and skin [122, 123] and have been shown to have good application prospects [124]. Odontogenic MSCs from third molars, orthodontic teeth, and deciduous teeth have been applied to dentin, periodontal tissue, dental pulp tissue, jaw defect repair, and other in vivo and in vitro studies and have been shown to have good regeneration ability [125].

Due to the same source of dental pulp tissue, E-MSCs have incomparable advantages in the field of dental pulp regeneration compared with M-MSCs, which is also a research hotspot of clinical application of E-MSCs. The majority of tooth loss is due to dental caries and root fractures. At present, root canal therapy is still the main treatment for pulpitis. However, the risk of root fracture is greatly increased due to the lack of nutrition from the pulp of the tooth after endodontic treatment. Therefore, the regenerative restoration of dental pulp has become the goal of functional tooth restoration. Gronthos et al. [8] first demonstrated the ability of DPSCs to differentiate into odontoblasts in 2000. SHEDs can be injected into the dental pulp cavity using injectable scaffold materials, which can not only maintain the nerve activity of dental pulp but also reconstruct the vascularized dental pulp tissue and have the ability to differentiate into odontoblasts [126].

Pulp regeneration of pulpless teeth has always been a dream of dentists and researchers. However, there are still many problems, including the longtime pulp regeneration and the use of scaffold materials that increase the risk of inflammation and infection. The dental pulp regeneration therapy technology established by scaffold-free 3D DPSC constructs avoids the potential problems caused by scaffold materials in transplanted pulp-like tissues [126]. Histological analysis showed that the transplanted DPSC constructs were differentiated into odontoblast-like cells at the site of contact with dentin and were able to form a vascular pulp-like tissue without the need for scaffolds or growth factors. The establishment and development of this technique suggest that the transplantation of DPSCs holds promise for the regeneration of pulp tissue in pulpless teeth.

Odontogenic MSCs also have a strong immunomodulatory effect. They can induce immune tolerance and reduce tissue damage caused by inflammatory reactions, which is conducive to the recovery and prognosis of damaged tissues. They have been applied to the immunomodulatory treatment of a variety of immune system diseases, such as systemic lupus erythematosus, colitis, and multiple sclerosis. After receiving stem cells or their secretions, symptoms related to these diseases can be alleviated. DPSCs are also expected to be able to treat type 2 diabetes and rheumatoid arthritis [127].

By May 2023, more than 12,000 clinical trials of MSCs had been retrieved from the ClinicalTrials.gov website. Internationally, approved MSC drugs have been listed in the United States, South Korea, Japan, and the European Union, and stem cell therapy has become a reality. Although E-MSCs have good biological properties and immunomodulatory ability, it is worth considering that their clinical application is far from that of M-MSCs. According to incomplete statistics, there are 11 MSC drugs approved for marketing worldwide (Table 4), including the United States (1), the European Union (2), Japan (3), South Korea (4), India (1), Australia (1), and Canada (1). From a review of the clinical trials obtained from the ClinicalTrials.gov website, we discovered that all the seed cells of stem cell drugs approved for clinical application are from the mesoderm, and no stem cell drugs from the ectoderm have been listed anywhere in the world.

What is the reason for this? We hypothesized that although the biological properties of E-MSCs are more suitable for tissue engineering, researchers have spent a relatively short amount of time researching them. BMSCs were first discovered 50 years ago, but the earliest E-MSCs were discovered approximately 20 years ago. Compared with MSCs from other sources, such as BMSCs and ADSCs, the foundation of E-MSC research is not strong enough, and relevant supporting research is not sufficient. It will take more time to study the biological properties and immune regulation characteristics of E-MSCs. Second, due to the short research time and imperfect supporting conditions, different researchers have different methods to isolate and culture E-MSCs. Different culture conditions, such as serum, cell inoculation density, and oxygen partial pressure, may affect cell proliferation and differentiation potential [128, 129]. Therefore, it is necessary to formulate an international unified standard process for the isolation, extraction, identification, and culture of E-MSCs. The age of donors also affects the proliferation and differentiation potential of MSCs. Studies have shown that MSCs from young donors show less damage and better proliferation [130].

In the field of tissue engineering, scaffold material is an indispensable factor. It can provide an environment for MSCs to perform their functions and is conducive to the further development of the therapeutic role of MSCs. The scaffold material for E-MSCs can improve the therapeutic effect of stem cells, and there is relatively little research in this field, which may also be one of the factors that hinders the further application of E-MSCs in regenerative medicine.

## 6. Summary

Both E-MSCs and M-MSCs have good self-renewal and multidirectional differentiation potential; are convenient and safe to extract, expand, and preserve; and have fewer ethical concerns. They are potential seed cells for tissue regeneration, repair, and clinical treatment in the future. However, the clinical application of E-MSCs is still limited. Research on the biological role, mechanism, and regulation after entering the host and differentiation into other tissues is still in the initial stage.

E-MSCs are valuable resources for regenerative medicine. The excellent differentiation potential of E-MSCs provides a new opportunity for the development of different research fields such as metabolic diseases, tumors, and injury repair. DPSC culture technology based on tissue engineering 3D scaffolds has great potential in dental pulp tissue regeneration. It is worth mentioning that the advantages of E-MSCs in neuronal differentiation are helpful for the research of many neurodegenerative diseases, such as Alzheimer's disease, Parkinson's disease, TBI, and peripheral nerve injury. E-MSC transplantation may become an effective treatment for restoring neurological function.

Further development of materials, science, molecular biology, and tissue engineering technology combined with increased understanding of the biological properties of MSCs from different germ layers will promote the clinical application of E-MSCs. And E-MSCs are expected to become a mature clinical technology and have a bright application prospect in the field of regenerative medicine, creating new alternative treatment options for a variety of diseases.

## Figures and Tables

**Table 1:** Extraction, discrimination, and culture of E-MSCs.

MSC type	First found time	Source	Extraction approach		Culture medium	Marker	Reference
DPSCs	2002	Pulp of human third molars	DPSC-ED	(1) Tooth surfaces were cleaned and cut around the cementum-enamel junction by using sterilized dental fissure burs to reveal the pulp chamber (2) Rinse with culture medium and cut dental pulp tissue into small pieces of 1-2 mm^3^ (3) Pulp fragments were digested in a solution of 3 mg/ml collagenase type I and 4 mg/ml dispase II for 1 h at 37°C (4) After digestion, it was centrifuged at a speed of 300 g, and the resuspended tissue fragments were passed through 70 *μ*M cell filter (5) The cells were cultured in 6-well plates in standard DPSC medium. All cell cultures were incubated at 37°C in 5% CO^2^	Alpha modification of Eagle's medium supplemented with 10% FBS+100 *μ*M L-ascorbic acid 2-phosphate+2 mM L-glutamine+100 U/ml penicillin+100 *μ*g/ml streptomycin	Positive: CD29, CD44, CD49f, CD73, CD81, CD90, CD105, and CD146 Negative: CD14, CD19, CD34, CD45, Stro-1, and HLA-DR	[8, 14–19]
			DPSC-OG	(1) After disinfecting the tooth surface, mechanically break the tooth and gently separate the dental pulp with tweezers (there is no need to drill, because it may adversely affect the viability of dental pulp stem cells) (2) Rinse with culture medium and cut dental pulp tissue into small pieces of 1-2 mm^3^ (3) Pulp pieces were cultured in 6-well plates in standard DPSC medium. All cell cultures were incubated at 37°C in 5% CO_2_			

DFSCs	2005	Normal human impacted third molars	(1) Normal human impacted third molars were surgically removed and collected. Attached dental follicles were separated from the mineralized tooth (2) The surfaces of the follicle tissues were cleaned and minced by using a sterilized scalpel (dental papilla tissue was discarded) (3) Tissues were digested in a solution of 3 mg/ml collagenase type I and 4 mg/ml dispase II for 1 h at 37°C (4) Minced and digested tissues of dental follicle explants were seeded into 60 mm plates or T25 flasks in media at 37°C in 5% CO_2_ in a humidified atmosphere		Alpha modification of Eagle's medium supplemented with 10% FBS+100 *μ*M L-ascorbic acid 2-phosphate+2 mM L-glutamine+100 units/ml penicillin+100 *μ*g/ml streptomycin	Positive: CD105, CD29, CD90, and CD73 Negative: CD45, CD19, HLA-DR, CD14, and CD34	[9, 20–23]

SCAPs	2006	Normal human impacted third molars	(1) Root apical papilla was gently separated from the surface of the root (2) Tissues were minced and digested in a solution of 3 mg/ml collagenase type I and 4 mg/ml dispase for 30 minutes at 37°C (3) Single cell suspensions of SCAP were obtained by passing through a 70 *μ*M strainer (4) Minced and digested tissues of dental follicle explants were seeded into 60 mm plates or T25 flasks in media at 37°C in 5% CO_2_ in a humidified atmosphere		Alpha modification of Eagle's medium supplemented with 15% FBS+100 *μ*M L-ascorbic acid 2-phosphate+2 mM L-glutamine+100 U/ml penicillin+100 *μ*g/ml streptomycin	Positive: STRO-1, CD24, CD29, CD73, CD90, CD105, CD106, CD146, CD166, and ALP Negative: CD34, CD45, CD18, and CD150	[10]
		Minipigs: canine	(1) The canines of Wuzhishan minipigs were extracted, and the root apical papilla was gently separated from the surface of the root (2) Apical papilla was minced and digested in a solution of 3 mg/ml collagenase type I and 4 mg/ml dispase for 30 minutes at 37°C (3) Then passed through a 70 *μ*m strainer to obtain a single cell suspension and seeded into 25 cm^2^ culture flasks containing an basic medium			Positive:STRO-1, CD146, CD24	[24]

SHEDs	2003	Intact caries free primary teeth	(1) The pulp was separated from a remnant crown under strict aseptic conditions (2) The pulp was minced by using a sterilized scalpel (3) Tissues were digested in a solution of 3 mg/ml collagenase type I and 4 mg/ml dispase for 30 minutes at 37°C (4) Then seeded into 25 cm^2^ culture flasks containing an basic medium		Dulbecco's modified Eagle's medium supplemented with 10% FBS+100 U/ml penicillin+100 *μ*g/ml streptomycin	Positive: STRO-1, STRO-3, CD13, CD29, CD44, CD73, CD90, CD105, CD106, CD166, CD271, CD146, ALP, MEPE, bFGF, and endostatin Negative: CD3, CD8, CD11b (or CD14), CD15, CD19 (or CD79*α*), CD33, CD34, CD45, CD71, CD117, and HLA-DR	[14, 25–27]

PDLSCs	2004	Normal impacted third molars	(1) PDL was gently separated from the surface of the root (2) Tissues was minced and digested in a solution of 3 mg/ml collagenase type I and 4 mg/ml dispase for 1 hour at 37°C (3) Single cell suspensions of PDLSCs were obtained by passing through a 70 *μ*M strainer (4) Minced and digested tissues were seeded into 60 mm plates or T25 flasks in media at 37°C in 5% CO_2_ in a humidified atmosphere		Alpha modification of Eagle's medium supplemented with 10% FBS+100 *μ*M L-ascorbic acid 2-phosphate+2 mM L-glutamine+100 units/ml penicillin+100 *μ*g/ml streptomycin	Positive: CD105, CD29, CD90, and CD73 Negative: CD45, CD19, HLA-DR, CD14, and CD34	[11, 22, 28, 29]

GMSCs	2009	Attached keratinized gingival tissues	(1) The tissues were deepithelialized and minced into 1–2 mm^2^ fragments (2) The minced tissues were digested in 2 mg/ml collagenase and 1 mg/ml dispase for 30 min (3) After discarding the first digested cell suspension, the tissues were digested in the same solution for 90 min at 37°C (4) Single cell suspensions of GMSCs were obtained by passing through a 70 *μ*M strainer (5) Minced and digested tissues were seeded into 60 mm plates or T25 flasks in media at 37°C in 5% CO_2_ in a humidified atmosphere		Alpha modification of Eagle's medium containing 15% FBS+100 U/ml penicillin+100 *μ*g/ml streptomycin +200 mM L-glutamine +10 mM ascorbic acid 2-hosphate	Positive: CD44, CD73, CD90, CD105, SSEA-4, STRO-1, CD146, CD166, and CD271 Negative: CD14, CD45, CD34, and CD19	[30]

OMSCs	2005	Human upper middle turbinates	(1) All biopsies were collected on ice in Hanks' balanced salt solution containing penicillin (100 U/ml), streptomycin (100 mg/ml), and Fungizone (amphotericin B, 1.25 mg/ml) (2) After being minced with a scalpel blade, the tissue was digested using 1.33% collagenase for 20 min (3) Then, the tissues were incubation with DNAse to reduce cell clumping (0.04 mg/ml bovine pancreas DNAse, 3.0 mg/ml bovine serum albumin-fraction A in L15) (4) Cells were mechanically dissociated by pipetting and then triturating through a 23G needle and centrifuged at 1200 rpm for 5 min, and the pellet resuspended in low-glucose Dulbecco's modified Eagle's medium		Low-glucose Dulbecco's modified Eagle's medium supplemented with 10% FBS+100 U/ml penicillin+100 *μ*g/ml streptomycin	Positive: CD90, CD54, CD105, CD73, nestin, CD166, and p75NTR Negative: STRO-1	[13]

JMSCs	2006	Human undergoing orthognathic surgery	The resected bone mass was cut into small fragments (<1 mm^3^) and cultured in T25 flasks with *α*-MEM containing 10% FBS in a 37°C humidified incubator with a 5% CO^2^ atmosphere		Alpha modification of Eagle's medium containing 10% FBS+100 U/ml penicillin+100 *μ*g/ml streptomycin	Positive: CD73, CD90, and CD105 Negative: CD34, CD11b, CD19, CD45, and HLA-DR	[31–34]

**Table 2:** The multiple differentiation potential and the derivation of ectodermal MSCs.

Cell type	PD	Multipotentiality	Source	Reference
DPSCs	60-70	Osteogenic	Human impacted third molar (age 18–22 years old)	[49]
			Rat	[50]
		Angiogenic	Human impacted third molar (age 18-25 years)	[51]
		Adipogenic	Supernumerary tooth (female, 8 years, and male, 12 years)	[52]
		Neurogenic	Human impacted third molars (age 20-30 years)	[53]
			Human impacted third molars	[54]
			Human impacted third molars (age 18–22 years)	[55]
			Human impacted third molars	[56]
		Dentin/pulp-like	Human impacted third molars (age 15-25 years)	[57]
			Human impacted third molar (11 years old)	[58]

DFSCs	—	Chondrogenesis	Rat	[59]
			Healthy children (age 6-12 years)	[60]
		Osteogenic	Rat	[61]
			Healthy children (age 6-12 years)	[60]
		Adipogenic	Healthy children (age 6-12 years)	[60]
			Human impacted third molar (female, 22 years)	[52]
		Neurogenic	Impacted third molar (age 18–22 years)	[55]

SCAPs	70	Angiogenesis	Human impacted third molars (age 12-15 years)	[62]
		Neurogenic	Human impacted third molars (age 18-22 years)	[55]
		Osteo/dentinogenic	Human impacted third molars (age 12-15 years)	[63]

SHEDs	<140	Neurogenic	Healthy children (age 7-8 years)	[64]
		Adipogenic	Healthy children (age 7-8 years)	[64]
		Osteo/dentinogenic	Healthy children (age 7-8 years)	[64]
		Chondrogenesis	Healthy children (age 7-8 years)	[64]
		Angiogenesis	Healthy children (age 7-8 years)	[65]

PDLSCs	—	Adipogenic	Supernumerary tooth (male, 12 years)	[52]
		Osteogenesis	Human periodontal ligament	[66]
				[67]
				[68]
		Angiogenesis	Human impacted third molars	[69]
		Adipogenic	Human periodontal ligament	[70]
		Chondrogenesis	Human periodontal ligament	[70]

GMSCs	—	Adipogenic	Human gingiva	[12]
		Chondrogenesis	Human gingiva	[12]
		Osteogenesis	Human gingiva	[12]
			Human gingiva	[71]
			Human gingiva (16 to 22 years old)	[72]
		Angiogenesis	Human gingiva	[73]
		Neurogenic	Human gingiva (20 to 40 years old)	[74]

OMSCs	—	Neurogenic	Rat	[75]
			Human olfactory mucosa	[76]
		Osteogenesis	Human olfactory mucosa	[76]
				[77]

JMSCs	50-60	Osteogenesis	Mouse jaw bone	[78]
				[35]
		Adipogenic	Mouse jaw bone	[35]
		Chondrogenesis	Mouse jaw bone	[35]

Abbreviation: PD: population doubling.

**Table 3:** The function of E-MSCs in mediating immune cells.

Immune cell type	E-MSC functions
PBMCs	Inhibiting PBMC proliferation
DCs	Inhibiting DC differentiation and maturation
Macrophage	Activating M2 macrophage polarization in general; activating M1 macrophage polarization in specific microenvironment
Mast cells	Inhibiting mast cell exocytosis
T cell	Inhibiting T cell proliferation, differentiation, and apoptosis
B cell	Inhibiting B cell exocytosis

Abbreviation: PBMCs: peripheral blood mononuclear cells; DCs: dendritic cells.

**Table 4:** 11 stem cell therapeutic drugs approved for marketing worldwide.

Country	Trade name	Cell type	Indication	Approved time
The United States	Prochymal	Bone marrow mesenchymal stem cells	Graft versus host disease (GVHD), Crohn's disease	2010.05

The European Union	Stempeucel	Bone marrow mesenchymal stem cells	Thromboangiitis obliterans	2015.06
	Alofisel	Adipose-derived mesenchymal stem cells	Crohn's disease with complex perianal fistula	2018.03

South Korea	Cell gram	Bone marrow mesenchymal stem cells	Acute myocardial infarction	2011.07
	Cartistem	Umbilical cord blood mesenchymal stem cells	Degenerative arthritis and knee cartilage injury	2012.01
	Cuepistem	Adipose-derived mesenchymal stem cells	Complex Crohn's disease complicated with anal fistula	2012.01
	NeuroNATA-R	Bone marrow mesenchymal stem cells	Amyotrophic lateral sclerosis, motor neuron disease	2014.07

Canada	Prochymal	Bone marrow mesenchymal stem cells	Graft versus host disease (GVHD) in children	2012.05

Australia	MPC	Autologous mesenchymal precursor cells	Repair of damaged bone tissue	2010.07

Japan	Temcell	Bone marrow mesenchymal stem cells	Graft versus host disease (GVHD)	2016.02
	RNL-Astrostem	Adipose-derived mesenchymal stem cells	Alzheimer's disease	2018.04
	Stemirac	Bone marrow mesenchymal stem cells	Spinal cord injury	2018.12

India	Stempeucel	Bone marrow mesenchymal stem cells	Severe lower limb ischemia caused by Burger's disease	2017

## Data Availability

All the data indicated in this study are available upon request by contacting the corresponding authors.
